# Recurring Temporal Association Between Influenza A Epidemics and Subsequent Influenza B Dominance in Post‐Pandemic Korea, 2022−2026

**DOI:** 10.1002/jmv.71169

**Published:** 2026-09-24

**Authors:** Yoon Lee, Yoonsun Yoon, Ji Eun Park, Won Keun Kim, Youn Young Choi, Yun‐Kyung Kim, Changmin Kang, Young Yoo, Young June Choe

**Affiliations:** ^1^ Department of Pediatrics, Korea University Anam Hospital Korea University College of Medicine Seoul Republic of Korea; ^2^ Department of Pediatrics, Korea University Guro Hospital Korea University College of Medicine Seoul Republic of Korea; ^3^ Global Child Health Institute Korea University Seoul Republic of Korea; ^4^ Department of Microbiology Hallym University College of Medicine Chuncheon Republic of Korea; ^5^ Department of Pediatrics, Korea University Ansan Hospital Korea University College of Medicine Seoul Republic of Korea; ^6^ Allergy and Immunology Center Korea University Seoul Republic of Korea

**Keywords:** influenza B, influenza surveillance, Korea, post‐pandemic, seasonal dominance

## Abstract

Post‐pandemic influenza dynamics have shown unusual patterns globally. We investigated the re‐emergence and recurrent seasonal dominance of influenza B in Korea across three post‐pandemic seasons (2022−2026). We analyzed 157 consecutive weeks of national sentinel surveillance data (K‐SenS, Korea Disease Control and Prevention Agency), covering September 2022 to August 2025, plus preliminary data through Week 10 of 2026. Influenza B predominance was defined as weeks with > 50% influenza B among total influenza detections; a sensitivity analysis required overall influenza activity > 5%. Influenza B activity remained minimal during the 2022−23 season (mean proportion 3.3%; peak 25.3%) and never reached predominance. Two complete predominance episodes followed influenza A epidemics: after the 2023−24 epidemic (co‐circulating A/H1N1pdm09 and A/H3N2; peak ILI 61.3/1000), B predominance began January 2024 and persisted 23 weeks (peak 100%); after the 2024−25A/H1N1pdm09‐dominant epidemic (peak ILI 99.8/1000), it began February 2025 and accumulated 20 weeks over two periods with a brief interruption (peak 100%). Onset occurred 5−8 weeks after the influenza A peak by ILI (6−8 weeks virologically) in all three episodes, unaffected by threshold choice. A third episode following the 2025−26A/H3N2 epidemic was ongoing as of March 2026 (onset 7 weeks after the A peak). In post‐pandemic Korea, influenza B repeatedly established seasonal dominance 5−8 weeks after influenza A epidemics across three consecutive seasons. This recurring temporal pattern may have implications for influenza surveillance, clinical preparedness, and vaccine strategy, though its underlying mechanism remains unestablished.

## 1 Introduction

Influenza B viruses have historically co‐circulated with influenza A, contributing substantially to seasonal influenza burden worldwide [1]. Unlike influenza A, influenza B infects only humans and is not subject to the same degree of antigenic variation, yet it causes significant morbidity, particularly in children and young adults [2]. Two distinct lineages of influenza B—B/Victoria and B/Yamagata—have co‐existed globally for decades, necessitating quadrivalent influenza vaccine formulations to provide coverage against both lineages [3].

The COVID‐19 pandemic fundamentally altered the epidemiology of seasonal respiratory viruses. Non‐pharmaceutical interventions (NPIs) implemented globally from early 2020—including mask mandates, school closures, and international travel restrictions—suppressed influenza circulation to historically low levels [4]. Influenza B was particularly affected: B/Yamagata lineage has not been detected since October 2020 and was formally declared extinct by the World Health Organization (WHO) in February 2024, leading to its removal from seasonal influenza vaccine compositions [5]. Meanwhile, the B/Victoria lineage also experienced a prolonged period of near‐complete absence during the pandemic years.

Following the relaxation of COVID‐19 pandemic control measures from 2022 onward, influenza viruses re‐emerged globally in a pattern distinct from pre‐pandemic seasonality [6]. Post‐pandemic influenza dynamics have been characterized by unusual timing, intensity, and subtype distributions. Several countries have reported atypical influenza A epidemics and shifts in age‐specific burden [7]. However, the post‐pandemic trajectory of influenza B, particularly the pattern of its re‐emergence and subsequent relationship with influenza A, has not been well characterized.

Korea provides an informative setting in which to examine post‐pandemic influenza dynamics. The Korea Sentinel Surveillance (K‐SenS) system, operated by the Korea Disease Control and Prevention Agency (KDCA), has maintained continuous weekly virological and clinical surveillance since before the pandemic, enabling longitudinal analysis across the pandemic transition period [8]. Korea also experienced a relatively sharp NPI relaxation in 2022, with a rapid return of respiratory virus circulation [9].

In this study, we analyzed 157 consecutive weeks of national sentinel surveillance data spanning September 2022 through August 2025 to characterize the re‐emergence and recurrent seasonal dominance of influenza B in Korea across three complete post‐pandemic influenza seasons. We hypothesized that influenza B re‐emergence would follow a recurring temporal pattern relative to influenza A epidemic peaks, and examined this relationship quantitatively.

## 2 Methods

### 2.1 Study Design and Data Source

We conducted a retrospective descriptive analysis of national sentinel surveillance data from the K‐SenS system, operated by the KDCA. K‐SenS collects weekly respiratory illness data from approximately 200 sentinel primary care clinics and 200 sentinel hospitals distributed nationwide. Data from weekly surveillance reports were extracted for the period from Week 36 of 2022 (September 4, 2022) through Week 35 of 2025 (August 30, 2025), comprising 157 consecutive surveillance weeks spanning three complete influenza seasons (2022–23, 2023–24, and 2024–25). Preliminary data from the 2025–26 season through Week 10 of 2026 (March 7, 2026) were additionally examined to assess for emerging patterns.

### 2.2 Surveillance Variables

The following weekly indicators were extracted: (1) influenza‐like illness (ILI) rates per 1000 outpatient visits; (2) influenza virological data, including total influenza detection rate (%) and subtype‐specific detection rates for influenza A/H1N1pdm09, A/H3N2, and influenza B; (3) acute respiratory illness (ARI) hospitalization counts, including total ARI, influenza‐associated, and severe acute respiratory illness (SARI) inpatients; and (4) detection rates of co‐circulating respiratory viruses, including rhinovirus, respiratory syncytial virus (RSV), human metapneumovirus (hMPV), human coronavirus (HCoV), parainfluenza virus, and SARS‐CoV‐2.

All influenza B detections were classified as influenza B without lineage designation, as K‐SenS reports do not routinely distinguish between B/Victoria and B/Yamagata lineages. Given that B/Yamagata lineage has not been detected globally since October 2020 and was declared extinct by WHO in February 2024, circulating influenza B during the study period is presumed to represent B/Victoria lineage; however, lineage‐level confirmation was not performed.

### 2.3 Definitions

Influenza B predominance was defined as any surveillance week in which influenza B accounted for more than 50% of total influenza detections, restricted to weeks with an influenza detection rate greater than 0%; this operational threshold identifies weeks in which a majority of circulating influenza is type B, but can be met by a small absolute number of detections when overall influenza activity is low. To address this, a sensitivity analysis was additionally performed requiring a minimum overall influenza detection rate of > 5% for a week to qualify as B‐predominant. The onset of influenza B predominance was defined as the first week meeting the primary criterion within each influenza season; onset timing was compared across the primary and sensitivity definitions. The inter‐epidemic interval was defined as the number of weeks elapsed between the week of peak ILI during the preceding influenza A epidemic and the week of influenza B predominance onset. Because ILI is not an influenza A‐specific indicator, this interval was additionally recalculated using the week of peak virological influenza A detection rate (the sum of subtype‐specific detection rates) for each season as an alternative epidemic peak metric. The influenza epidemic period for each season was defined as consecutive weeks during which the ILI rate met or exceeded the nationally defined epidemic threshold (2022−23: 4.9; 2023−24: 6.5; 2024−25: 8.6; 2025−26: 9.1 per 1000 outpatient visits).

### 2.4 Statistical Analysis

Descriptive statistics were used to characterize influenza detection rates, ILI levels, and hospitalization burden by season and by epidemic phase. Spearman's rank correlation coefficients were calculated between weekly influenza B detection rates and detection rates of each co‐circulating respiratory virus at lags of 0−4 weeks (virus activity leading influenza B by the stated number of weeks); the contemporaneous (lag 0) correlation is reported in the main text, and the full set of lag‐specific results is provided in Table S1. Because these correlations involve multiple viruses and lags without adjustment for multiple comparisons, and because consecutive weekly surveillance observations are not independent and may share seasonal structure, these analyses are exploratory and were interpreted with caution rather than as tests of a pre‐specified hypothesis. Cumulative influenza inpatient counts reported during influenza B predominance periods represent all laboratory‐confirmed influenza (A and B combined) hospitalizations occurring in those weeks, as K‐SenS hospitalization data are not stratified by influenza type. Figures were generated using R (version 4.3) with the ggplot2 package. All analyses were performed using Python (version 3.12).

### 2.5 Ethical Considerations

This study was based entirely on publicly available, de‐identified, aggregated surveillance data published by the KDCA. No individual patient data were accessed. Korea University Anam Hospital Institutional Review Board has reviewed and approved the protocol (IRB No. 2026AN0081).

## 3 Results

### 3.1 Overall Influenza Surveillance, 2022–2025

**Figure 1:**
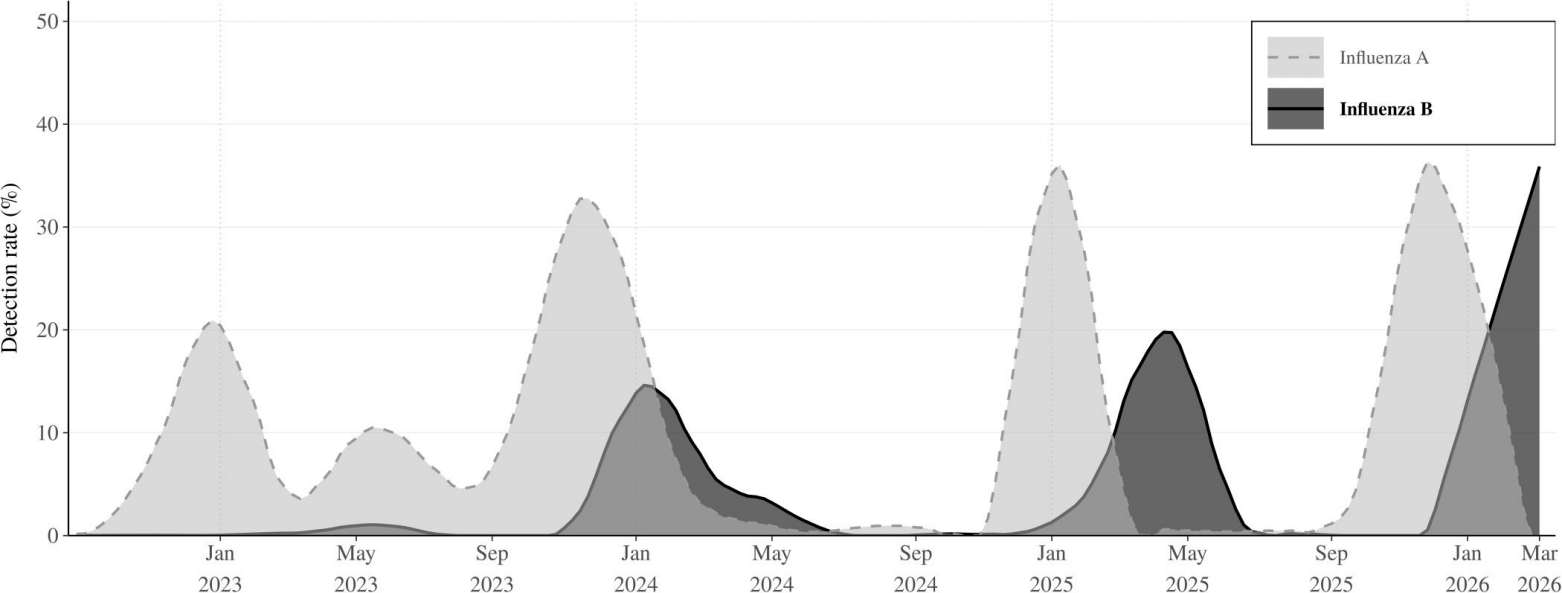
Weekly influenza A and influenza B detection rates in Korea, September 2022 through March 2026 (*n* = 184 surveillance weeks). (A) Shows stacked weekly detection rates with influenza A (dark gray) and influenza B (light gray) displayed as independent overlapping areas, with LOESS‐smoothed trend curves. (B) Shows the same data in a diverging format, with influenza A plotted above and influenza B below the zero axis, illustrating the relative shift in dominance between the two types across seasons. Dotted vertical lines indicate January of each year. Data source: Korea Sentinel Surveillance (K‐SenS) weekly reports, Korea Disease Control and Prevention Agency (KDCA).

A total of 157 surveillance weeks were analyzed across three complete post‐pandemic influenza seasons, with additional preliminary data from 27 weeks of the 2025–26 season (Table 1). The 2022–23 season was characterized by A/H3N2 dominance, with a peak ILI of 60.7 per 1000 outpatient visits in Week 53 of 2022 and a peak influenza detection rate of 31.2%. The 2023−24 season featured co‐circulation of A/H1N1pdm09 and A/H3N2, with a peak ILI of 61.3 per 1000 and peak influenza detection of 43.8%; A/H1N1pdm09 predominated during the initial epidemic weeks (W47‐49/2023: mean subtype‐specific detection 21.9% vs. 12.6% for A/H3N2), whereas A/H3N2 became dominant from Week 50 of 2023 onward and over the epidemic period as a whole. The 2024–25 season produced the highest influenza burden of the study period, with an A/H1N1pdm09‐dominant epidemic reaching a peak ILI of 99.8 per 1000—the highest recorded since the inception of K‐SenS—and a peak detection rate of 62.9%. The 2025–26 season, dominated by A/H3N2, reached a peak ILI of 70.9 per 1000 as of the data cutoff (Figure 1).

**Table 1:** Influenza surveillance characteristics by season, Korea 2022–2026.

Characteristic	Influenza season			
	2022–23	2023–24	2024–25	2025–26^a^
Season overview				
Dominant influenza A subtype	A/H3N2	A/H1N1pdm09→A/H3N2 (co‐circulating)^b^	A/H1N1pdm09	A/H3N2
Observation period	W36/2022–W35/2023 (53 weeks)	W36/2023–W35/2024 (52 weeks)	W36/2024–W35/2025 (52 weeks)	W36/2025–W10/2026 (27 weeks)
Epidemic period^c^	W45/2022–W32/2023 (38 weeks)	W38/2023–W15/2024 (27 weeks)	W49/2024–W20/2025 (24 weeks)	W43/2025–W10/2026 (20 weeks)
Influenza‐like illness (ILI, per 1000 outpatient visits)				
Peak ILI	60.7	61.3	99.8	70.9
Week of peak ILI	W53/2022	W49/2023	W1/2025	W47/2025
Mean ILI during epidemic period	18.1	19.2	15.0	35.2
Virological surveillance				
Peak influenza detection rate (%)	31.2	43.8	62.9	45.0
Mean influenza detection rate (%)	8.7	14.4	12.3	27.0
Peak influenza A detection rate (%)	31.2	37.3	61.1	44.7
Peak influenza B detection rate (%)	2.0	19.6	28.3	36.0
Influenza B proportion among all influenza detections				
Mean B proportion (%)^d^	3.3	34.9	39.7	30.4
Peak B proportion (%)	25.3	100	100	94.9
Weeks with B proportion > 50% (*n*)	0	23	20	9
First week of influenza B predominance^e^	Not observed	W2/2024	W9/2025	W2/2026
Acute respiratory illness hospitalization				
Cumulative ARI inpatients (*n*)	58 468	94 498	75 423	35 528
Cumulative influenza inpatients (*n*)	6101	12 875	9246	8560
Peak weekly influenza inpatients (*n*)	472	1101	1627	705
Cumulative SARI inpatients (*n*)	12 163	12 051	11 130	6550

*Note:* Data source: Korea Sentinel Surveillance (K‐SenS) weekly reports, Korea Disease Control and Prevention Agency (KDCA).

Abbreviations: ARI, acute respiratory illness; ILI, influenza‐like illness; SARI, severe acute respiratory illness.

^a^ 2025–26 season data through W10/2026 (March 7, 2026); data are preliminary.

^b^ A/H1N1pdm09 predominated during the initial epidemic weeks of the 2023–24 season (W47–49/2023: mean subtype‐specific detection rate 21.9% for A/H1N1pdm09 vs. 12.6% for A/H3N2), whereas A/H3N2 became the dominant subtype from week 50 of 2023 onward and over the epidemic period as a whole. Co‐circulation of both subtypes continued through the post‐epidemic spring wave (W2–W20/2024), during which influenza B became predominant.

^c^ Epidemic period is defined as consecutive weeks with ILI ≥ national epidemic threshold (2022–23: 4.9; 2023–24: 6.5; 2024–25: 8.6; 2025–26: 9.1 per 1000 outpatient visits).

^d^ Calculated among weeks with an influenza detection rate > 0%.

^e^ Defined as the first week in which influenza B accounted for > 50% of total influenza detections.

### 3.2 Re‐Emergence of Influenza B

Influenza B activity remained minimal during the 2022−23 season, with a mean proportion of 3.3% among influenza detections and a maximum of 25.3% observed transiently in isolated weeks; this level did not meet the predominance threshold in any week. This represented a marked contrast to pre‐pandemic seasons and is consistent with sustained suppression of influenza B during the pandemic period. Influenza B first re‐emerged at low levels during spring 2023.

### 3.3 First Episode of Influenza B Predominance: 2023–24 Season

Following the 2023−24 epidemic, which peaked at ILI 61.3 per 1000 in Week 49 of 2023 (virological influenza A peak: Week 48 of 2023), the influenza B proportion began rising rapidly from early January 2024. Influenza B predominance was first established in Week 2 of 2024 (January 7−13, 2024), 5 weeks after the ILI peak (6 weeks after the virological influenza A peak), when influenza B accounted for 51.2% of total influenza detections (Table 2). B predominance persisted for 23 consecutive weeks (Week 2 through Week 24 of 2024, i.e., through approximately June 10−16, 2024), reaching a peak proportion of 100% during Weeks 17−20 of 2024. Under the sensitivity threshold requiring overall influenza activity > 5%, this episode spanned 15 weeks (Week 2 through Week 16 of 2024); the onset week was unchanged. Cumulative influenza inpatients (influenza A and B combined) during the B predominance period totaled 3476, with a peak of 726 influenza inpatients in a single week.

**Table 2:** Characteristics of recurrent Influenza B predominance events by season, Korea 2023–2026.

Characteristic	Influenza season		
	2023–24	2024–25	2025–26
Dominant A subtype	A/H1N1pdm09 (early)/A/H3N2 (late)^a^	A/H1N1pdm09	A/H3N2
ILI peak (/1000 outpatient visits)	61.3	99.8	70.9
Week of ILI peak	W49/2023	W1/2025	W47/2025
Week of virological influenza A peak	W48/2023	W1/2025	W47/2025
Weeks from ILI peak to B predominance onset	5	8	7
Weeks from virological A peak to B predominance onset	6	8	7
Influenza B proportion at onset (%)	51.2	67.6	52.5
Duration of B predominance, primary definition^b^	23 weeks (W2‐24/2024)	20 weeks, two periods^c^	≥ 9 weeks, ongoing^d^
Duration under sensitivity threshold (flu activity > 5%)^e^	15 weeks (W2‐16/2024)	13 weeks, two periods^f^	≥ 9 weeks, ongoing^d^

*Note:* Data source: Korea Sentinel Surveillance (K‐SenS), Korea Disease Control and Prevention Agency (KDCA).

Abbreviation: ILI, influenza‐like illness.

^a^ Co‐circulation of A/H1N1pdm09 and A/H3N2 was observed; A/H1N1pdm09 predominated during the initial epidemic weeks (W47‐49/2023), A/H3N2 predominated thereafter and over the epidemic period as a whole.

^b^ Defined as consecutive weeks with influenza B accounting for > 50% of total influenza detections, among weeks with any influenza detected.

^c^ W9‐23/2025 (15 weeks) and W25‐29/2025 (5 weeks), separated by a single week (W24/2025) with no influenza detected of any type.

^d^ Data through W10/2026 (March 7, 2026); the 2025−26 season is ongoing, and the full duration of this episode has not yet been observed.

^e^ Sensitivity definition additionally requires a total influenza detection rate > 5% in the same week.

^f^ W9‐20/2025 (12 weeks) and W23/2025 (1 week).

### 3.4 Second Episode of Influenza B Predominance: 2024–25 Season

The 2024−25 season was notable for an unprecedented A/H1N1pdm09 epidemic, with a peak ILI of 99.8 per 1000 outpatient visits in Week 1 of 2025 (also the virological influenza A peak)‐the highest recorded in the K‐SenS surveillance history. Despite the exceptional intensity of the preceding influenza A epidemic, influenza B followed a similar temporal pattern. Influenza B predominance was established in Week 9 of 2025 (February 23 to March 1, 2025), 8 weeks after the influenza A peak by either metric, with a B proportion of 67.6% at onset. Predominance accumulated 20 weeks in total, over two periods (Week 9 through Week 23, and Week 25 through Week 29 of 2025) separated by a single week (Week 24 of 2025) with no influenza detected; the peak B proportion of 100% occurred during Weeks 13−17 of 2025. Under the sensitivity threshold requiring overall influenza activity > 5%, this episode spanned 13 weeks (12 weeks from Week 9 through Week 20, plus one additional week at Week 23); the onset week was unchanged. Cumulative influenza inpatients (influenza A and B combined) during this period totaled 1407, with a peak of 176 in a single week.

### 3.5 Emerging Third Episode: 2025–26 Season

Following the 2025−26A/H3N2 epidemic (peak ILI 70.9 per 1000 in Week 47 of 2025, coinciding with the virological influenza A peak), influenza B again began rising, establishing predominance in Week 2 of 2026 (January 4−10, 2026), 7 weeks after the influenza A peak by either metric‐consistent with the pattern observed in the two preceding seasons. As of the data cutoff (March 7, 2026), B predominance had persisted for at least 9 consecutive weeks, with a peak B proportion of 94.9% in Week 9 of 2026, suggesting continuation of this recurring pattern into a third season.

### 3.6 Temporal Pattern of B Predominance Onset

**Figure 2:**
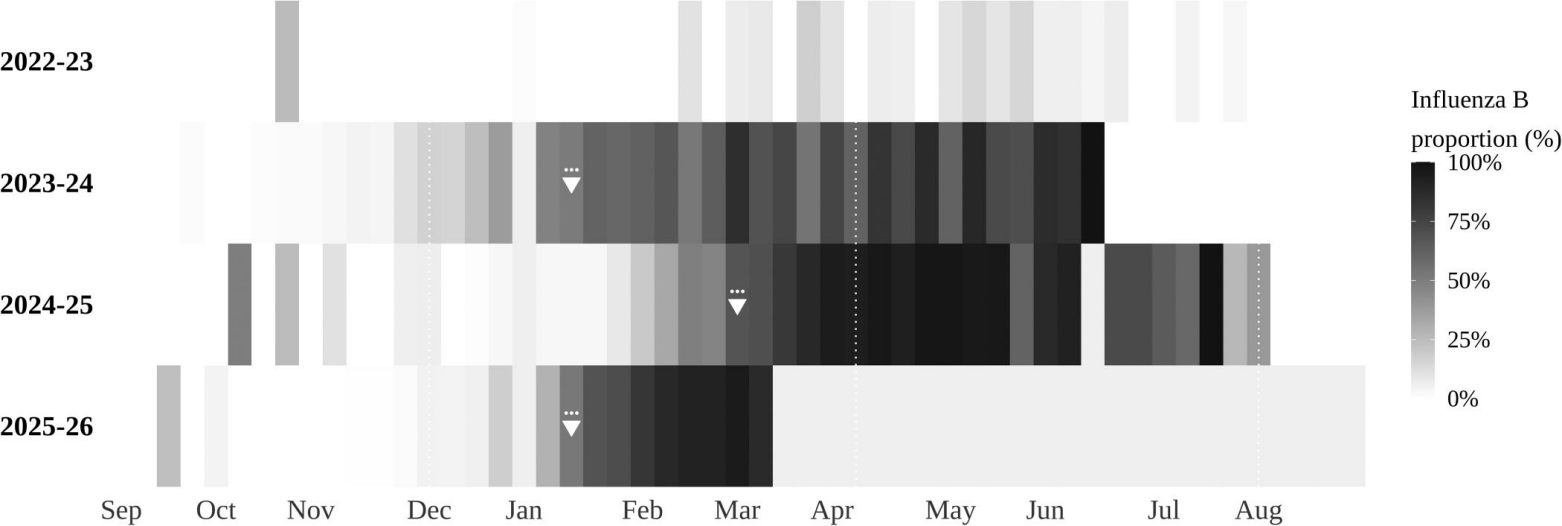
Heatmap of weekly influenza B proportion (percentage of influenza B among total influenza detections) across four post‐pandemic influenza seasons in Korea, aligned by season week (Week 1 = first week of September). Gray cells indicate weeks with no influenza of any type detected. White arrows indicate the first week in which influenza B accounted for > 50% of total influenza detections (influenza B predominance onset) in each respective season. For the 2024−25 season, the period of influenza B predominance was interrupted by a single week (Week 24 of 2025, shown as a gray cell) with no influenza detected; this interruption is reflected in Table 2. Data source: Korea Sentinel Surveillance (K‐SenS) weekly reports, Korea Disease Control and Prevention Agency (KDCA).

Across the two complete episodes and the emerging third, the interval from influenza A peak to influenza B predominance onset was 5, 8, and 7 weeks using the ILI‐based peak definition (range 5−8 weeks), and 6, 8, and 7 weeks using the virological influenza A peak definition (range 6−8 weeks); onset timing was essentially unchanged between the two definitions and was also unaffected by the > 5% activity sensitivity threshold. This temporal regularity was observed despite substantial differences in the intensity of the preceding influenza A epidemics (ILI peaks ranging from 61.3 to 99.8 per 1000) and in the dominant A subtype composition across seasons. Because all three onset events occurred in January or February, this pattern could also reflect the underlying seasonal ordering of influenza A and B circulation in Korea rather than a specific epidemiological interaction between the two viruses; this alternative explanation is considered further in the Discussion. The heatmap visualization of weekly B proportion aligned by season week illustrates the recurrent emergence of B predominance in the January to February period across the three affected seasons (Figure 2).

### 3.7 Co‐Circulating Respiratory Viruses

During periods of influenza B predominance, COVID‐19 detection rates were generally low (typically < 5%), consistent with the temporal displacement of influenza B predominance to spring months when SARS‐CoV‐2 activity was reduced. Conversely, the summer periods of influenza B absence (June to August) coincided with resurgence of COVID‐19 activity, reaching detection rates of 30%−43% during summer waves in 2024 and 2025 (Figure S1). Rhinovirus and parainfluenza virus showed inverse temporal relationships with influenza B, with higher detection rates observed during off‐season periods. Contemporaneous (lag 0) Spearman's correlation analysis revealed a significant positive association between influenza B detection rates and HCoV rates (rho = 0.61, *p* < 0.001, *n* = 167), and significant negative associations with parainfluenza (rho = −0.22, *p* = 0.005, *n* = 172) and COVID‐19 (rho = −0.40, *p* < 0.001, *n* = 145); correlations at lags of 1−4 weeks were of similar magnitude and direction (Table S1). Given the shared winter‐to‐spring seasonality of influenza B and HCoV, temporal autocorrelation in weekly surveillance data, and the exploratory, uncorrected nature of these comparisons, these associations should be interpreted as descriptive rather than as evidence of a direct virus−virus interaction.

## 4 Discussion

In this analysis of 157 weeks of national sentinel surveillance data, we identified a recurring temporal pattern in which influenza B seasonal dominance followed influenza A epidemics in two of three post‐pandemic seasons in Korea, with a third episode currently underway. Influenza B activity, which remained minimal during the 2022−23 season, subsequently reached predominance for 20−23 weeks in each of the following two complete seasons. In each of the three episodes, B predominance onset occurred 5−8 weeks after the preceding influenza A epidemic peak (ILI‐based) or 6−8 weeks (virological peak‐based), a timing that was consistent across differences in the dominant influenza A subtype and in the intensity of the preceding A epidemic, and was not sensitive to the choice of epidemic peak metric or predominance threshold.

The near‐absence of influenza B during the 2022−23 season is consistent with the global pattern of prolonged influenza B suppression during the COVID‐19 pandemic [10]. NPIs implemented during 2020−2022 preferentially suppressed influenza B over influenza A, likely due to differences in transmission dynamics [11, 12]. The formal declaration of B/Yamagata extinction by the WHO in 2024 underscores the depth of this suppression. One possible explanation for the subsequent re‐emergence of influenza B/Victoria in Korea from spring 2023 onward is a gradual recovery from an accumulated population‐level immunity debt during the pandemic years; however, as this study is based on aggregate surveillance data without direct immunological measurement, this remains a hypothesis rather than a demonstrated mechanism.

The 5−8 week interval between influenza A peaks and influenza B predominance onset is a key descriptive finding of this study. This regularity is compatible with, but does not establish, a direct mechanistic relationship between the two events. Possible explanations, which remain hypotheses rather than conclusions supported by this study design, include population‐level immune dynamics (e.g., waning cross‐reactive immunity to influenza B as influenza A activity declines) or an ecological‐niche effect whereby reduced influenza A competition allows influenza B to expand [13, 14]. Viral interference has also been proposed as a mechanism for temporal separation between influenza A and B circulation; however, this descriptive, aggregate‐level surveillance study cannot distinguish these mechanisms from a simpler explanation, namely that influenza A typically peaks earlier in winter while influenza B activity typically increases later in winter or spring for reasons unrelated to any specific interaction between the two viruses. Because all three B predominance onsets occurred in January or February, the observed pattern may in part reflect this underlying seasonal ordering rather than an epidemic‐specific effect of the preceding influenza A wave.

The 2024−25 influenza A/H1N1pdm09 epidemic produced the highest ILI peak recorded in K‐SenS surveillance history (99.8 per 1000 outpatient visits), representing an exceptional burden. Notably, influenza B nonetheless reached predominance within 8 weeks of this peak, similar to the other two seasons with substantially lower A epidemic intensity, suggesting that the observed timing is not simply a function of the size of the preceding influenza A epidemic [15]. Whether this reflects a shared underlying ecological or immunological process, or simply the seasonal timing of influenza B circulation each year, cannot be determined from this descriptive analysis [16].

The clinical implications of a recurring influenza B predominance phase are potentially important, particularly for pediatric populations. Influenza B is associated with a disproportionate burden in children and young adults, including rare but severe complications such as influenza B‐associated myositis and Reye syndrome‐like illness [17]. During the two complete B predominance episodes documented in this study, cumulative influenza (A and B combined) inpatients during B‐predominant weeks totaled 3476 and 1407, respectively; because K‐SenS hospitalization data are not stratified by influenza type, these figures cannot be interpreted as direct measures of influenza B‐specific clinical burden. With B/Yamagata lineage now extinct, all influenza B burden is presumed attributable to B/Victoria, and current trivalent vaccine compositions should provide adequate lineage coverage. If the recurring spring predominance pattern described here is confirmed in future seasons, the temporal concentration of influenza B following the winter influenza A epidemic may have implications for clinical preparedness, healthcare resource planning, and public health communication.

This study has several limitations. First, K‐SenS data represent sentinel surveillance rather than population‐based incidence, and detection rates reflect testing practices as well as true virus circulation. Second, influenza B lineage confirmation was not routinely performed, and we assumed all circulating influenza B represented B/Victoria based on WHO guidance; residual B/Yamagata activity cannot be formally excluded for the earliest weeks of the study period. Third, this is a descriptive surveillance study, based on only two complete and one ongoing episode, and cannot distinguish a specific epidemiological interaction between influenza A and B from the underlying seasonal ordering of the two viruses, nor can it support causal or mechanistic inferences about the drivers of the observed pattern. Fourth, the influenza B predominance threshold (> 50% of detections) can be met by a small absolute number of detections when overall influenza activity is low, as occurred during part of the 2024−25 episode; a sensitivity analysis using a minimum activity threshold yielded shorter but qualitatively similar episode durations, with unchanged onset timing. Fifth, the correlation analyses between influenza B and co‐circulating viruses did not correct for multiple comparisons across viruses and lags and did not account for temporal autocorrelation or shared seasonality, and should be regarded as exploratory. Sixth, cumulative hospitalization counts during B‐predominant weeks reflect all laboratory‐confirmed influenza (A and B) and cannot be restricted to influenza B specifically. Seventh, data for the 2025−26 season are preliminary, and the full extent of the third B predominance episode remains to be characterized. Finally, age‐disaggregated influenza B data were not available in K‐SenS weekly reports, limiting our ability to characterize age‐specific burden.

In conclusion, we describe a recurring temporal pattern of influenza B seasonal dominance in the weeks following influenza A epidemics in post‐pandemic Korea, with B predominance onset occurring 5−8 weeks after the influenza A peak in each of three consecutive seasons; this timing was robust to the choice of epidemic peak metric and predominance threshold. Whether this pattern reflects a specific epidemiological relationship between influenza A and B, or the seasonal ordering of the two viruses more generally, cannot be determined from this descriptive analysis. If confirmed in subsequent seasons, this pattern may have implications for seasonal influenza surveillance, clinical preparedness, and vaccine strategy. Ongoing surveillance will be essential to determine whether this pattern persists as population immunity continues to evolve in the post‐pandemic era.

## Author Contributions

**Yoon Lee:** data curation, formal analysis, writing – original draft. **Yoonsun Yoon:** data curation, formal analysis, writing–original draft. **Ji Eun Park:** investigation, data curation, writing – review and editing. **Won Keun Kim:** investigation, writing–review and editing. **Youn Young Choi:** investigation, writing – review and editing. **Yun‐Kyung Kim:** investigation, writing – review and editing. **Changmin Kang:** resources, writing – review and editing. **Young Yoo:** resources, writing – review and editing. **Young June Choe:** conceptualization, methodology, formal analysis, writing – original draft, writing – review and editing, supervision, project administration. All authors reviewed and approved the final version of the manuscript.

## Conflicts of Interest

The authors declare no conflicts of interest.

## Supporting information


Supporting File 1

Supporting File 2

## Data Availability

Surveillance data are publicly available from the Korea Disease Control and Prevention Agency (KDCA) weekly sentinel surveillance reports (https://www.kdca.go.kr).
