# Supplementary material for: Recurring Temporal Association Between Influenza A Epidemics and Subsequent Influenza B Dominance in Post‐Pandemic Korea, 2022−2026
Source: J Med Virol. 2026 Sep 24;98(10):e71169. doi: 10.1002/jmv.71169 (PMC13602433; doi:10.1002/jmv.71169)
Supplement: Supplementary file 1 [file JMV-98-e71169-s001.docx]

Supplementary Table 1. Spearman rank correlation coefficients between weekly influenza B detection rate and detection rates of co-circulating respiratory viruses, at lags of 0–4 weeks, Korea, 2022–2026.

| **Virus** | **Lag (weeks)** | **ρ** | **p-value** | **n** |
| --- | --- | --- | --- | --- |
| Human coronavirus (HCoV) | 0 | 0.61 | <0.001 | 167 |
|  | 1 | 0.61 | <0.001 | 166 |
|  | 2 | 0.61 | <0.001 | 165 |
|  | 3 | 0.64 | <0.001 | 164 |
|  | 4 | 0.60 | <0.001 | 163 |
| Parainfluenza virus | 0 | -0.22 | 0.005 | 172 |
|  | 1 | -0.26 | 0.001 | 171 |
|  | 2 | -0.31 | <0.001 | 170 |
|  | 3 | -0.35 | <0.001 | 169 |
|  | 4 | -0.40 | <0.001 | 168 |
| SARS-CoV-2 (COVID-19) | 0 | -0.40 | <0.001 | 145 |
|  | 1 | -0.44 | <0.001 | 144 |
|  | 2 | -0.46 | <0.001 | 143 |
|  | 3 | -0.48 | <0.001 | 142 |
|  | 4 | -0.47 | <0.001 | 141 |
| Rhinovirus | 0 | -0.11 | 0.164 | 178 |
|  | 1 | -0.09 | 0.230 | 177 |
|  | 2 | -0.11 | 0.159 | 176 |
|  | 3 | -0.11 | 0.153 | 175 |
|  | 4 | -0.10 | 0.207 | 174 |
| Respiratory syncytial virus (RSV) | 0 | 0.17 | 0.022 | 175 |
|  | 1 | 0.24 | 0.001 | 174 |
|  | 2 | 0.26 | <0.001 | 173 |
|  | 3 | 0.31 | <0.001 | 172 |
|  | 4 | 0.33 | <0.001 | 171 |
| Human metapneumovirus (HMPV) | 0 | 0.17 | 0.027 | 170 |
|  | 1 | 0.17 | 0.026 | 169 |
|  | 2 | 0.12 | 0.127 | 168 |
|  | 3 | 0.06 | 0.422 | 167 |
|  | 4 | 0.02 | 0.822 | 166 |
| Adenovirus | 0 | 0.02 | 0.770 | 171 |
|  | 1 | 0.03 | 0.722 | 170 |
|  | 2 | 0.01 | 0.870 | 169 |
|  | 3 | -0.02 | 0.849 | 168 |
|  | 4 | -0.01 | 0.943 | 167 |
| Bocavirus | 0 | -0.22 | 0.004 | 168 |
|  | 1 | -0.28 | <0.001 | 167 |
|  | 2 | -0.31 | <0.001 | 166 |
|  | 3 | -0.40 | <0.001 | 165 |
|  | 4 | -0.42 | <0.001 | 164 |

*Lag denotes the number of weeks by which the co-circulating virus detection rate leads the influenza B detection rate (e.g., lag 1 correlates the virus rate in week t−1 with the influenza B rate in week t). The contemporaneous (lag 0) values for HCoV, parainfluenza virus, and COVID-19 are also reported in the main text.*

*p-values are not adjusted for multiple comparisons across viruses and lags. Consecutive weekly surveillance observations are not independent, and several viruses share winter-to-spring seasonality with influenza B; these correlations are therefore exploratory and should be interpreted with caution rather than as evidence of a specific epidemiological relationship (see Discussion, Limitations).*

*HCoV, human coronavirus; RSV, respiratory syncytial virus; HMPV, human metapneumovirus.*
