# Supplementary material for: Recurring Temporal Association Between Influenza A Epidemics and Subsequent Influenza B Dominance in Post‐Pandemic Korea, 2022−2026
Source: J Med Virol. 2026 Sep 24;98(10):e71169. doi: 10.1002/jmv.71169 (PMC13602433; doi:10.1002/jmv.71169)
Supplement: Supplementary file 2 [file JMV-98-e71169-s002.docx]

Supplementary Figure 1. Weekly detection rates of co-circulating respiratory viruses in Korea, September 2022 through March 2026.

Shaded regions indicate periods of influenza B predominance (influenza B proportion >50%). Viruses shown include rhinovirus, respiratory syncytial virus (RSV), human metapneumovirus (HMPV), human coronavirus (HCoV), parainfluenza virus, and SARS-CoV-2 (COVID-19). Data source: Korea Sentinel Surveillance (K-SenS) weekly reports, Korea Disease Control and Prevention Agency (KDCA).
